# Subchondral Rafting Plate for the Treatment of Fragmented Articular Central Depression Tibial Plateau Fracture Patterns: Case Series and Technical Illustration

**DOI:** 10.7759/cureus.12740

**Published:** 2021-01-16

**Authors:** Vincenzo Giordano, Robinson E Pires, Kodi E Kojima, Sergei T Fischer, Peter V Giannoudis

**Affiliations:** 1 Serviço de Ortopedia e Traumatologia Prof. Nova Monteiro, Hospital Municipal Miguel Couto (HMMC), Rio de Janeiro, BRA; 2 Departamento de Ortopedia, Universidade Federal de Minas Gerais, Belo Horizonte, BRA; 3 Instituto de Ortopedia e Traumatologia, Hospital das Clinicas, Faculdade de Medicina, Universidade de Sao Paulo (HCFMUSP), São Paulo, BRA; 4 Departamento de Ortopedia, Hospital do Trabalhador, Universidade Federal do Paraná, Curitiba, BRA; 5 Trauma and Orthopaedics, Leeds Teaching Hospitals NHS Trust, Leeds, GBR

**Keywords:** tibial plateau, articular cartilage, subchondral bone, tibia fractures, subchondral rafting plate

## Abstract

Articular depression is a significant component of lateral tibial plateau fracture patterns. Current literature supports the use of subchondral rafting screws, either placed through a plate or not. However, articular comminution has been associated with increased articular subsidence despite an adequate screw-joint distance. We report four cases that underwent the subchondral rafting plate technique for fragmented articular central depression lateral tibial plateau fractures. Clinical and radiographic evaluations were performed at the last follow-up. The mean follow-up was 18 months. All patients healed the fracture without any significant articular subsidence or loss of reduction. This case study hints that this novel technique is a potentially safe and cost-effective strategy to be incorporated in the daily practice of the orthopedic trauma surgeon, especially in certain challenging circumstances when a salvage procedure is required due to lateral tibial plateau fracture malreduction and the unavailability of anatomically designed locking plates.

## Introduction

Tibial plateau fractures continue to attract great interest for both clinicians and scientists [1]. This occurs mainly due to various difficult surgical aspects such as the choice of approach, articular fracture reduction, and fixation strategies. In this scenario, articular central depression, a significant component of lateral condyle fracture patterns, is particularly challenging because of the high risk of secondary subsidence [2]. Current literature supports the use of subchondral rafting screws, either placed through a plate or not [2,3]. The addition of bone graft or other bone substitute is highly recommended to increase subchondral stiffness, although it was not found to be statistically significant in both experimental and clinical studies [2,4]. Moreover, complications after autografting have been reported in up to 22% of cases, including herniation, cosmetic defects, and chronic pain [4].

The basic concept of subchondral rafting involves the use of up to four 3.5-mm cortical screws placed within five millimeters of the tibial plateau subchondral bone in the axial plane to support subsequent re-displacement of the articular surface [2,4]. In a recent study, it was shown that fractures fixed with screws closer to the joint were correlated with less articular subsidence [5]. However, articular comminution has been associated with increased articular subsidence despite an adequate screw-joint distance [5].

Our hypothesis is that placement of wider support in the subchondral area of the tibial plateau could create a more stable construction for fragmented articular central depression fragments of the lateral tibial plateau, thus reducing the risk of subsidence. Thus, in the current study, we present the subchondral rafting plate technique for fragmented articular central depression tibial plateau fracture patterns.

## Technical report

Understanding the indication

Radiographic series of the knee, consisting of anteroposterior (AP), lateral, and 45º oblique views, and computed tomography (CT) scanning, including three-dimensional (3-D) reconstruction, are taken for all patients presenting with a tibial plateau fracture. Fracture characteristics, including fragment(s) morphology (shear and/or articular depression), loss of peripheral containment, and direction and amount of displacement are analyzed. Fractures are classified using the revisited Kfuri-Schatzker system [6], and the most appropriate treatment strategy is implemented.

Whenever present, the degree of comminution of the articular surface depressed area is estimated with a CT scan and the subchondral rafting plate technique is planned. The main indication for the technique is a fragmented articular central depression in lateral tibial plateau fracture patterns, especially in certain challenging circumstances when a salvage procedure is required due to lateral tibial plateau fracture malreduction.

Patient positioning and preparation for surgery

Patient positioning depends on the region (lateral and/or medial) and the quadrant (anterior and/or posterior) that needs to be elevated based on the Kfuri-Schatzker classification [6]. In general, articular depression is observed in the lateral region of the tibial plateau, either in unicondylar or bicondylar fracture patterns. Therefore, the supine position is used for anterolateral (AL) quadrant fractures and the prone position for posterolateral (PL) quadrant fractures.

A radiolucent surgical table is used, and a tourniquet is applied to the upper thigh but is not inflated during the procedure unless it is necessary. The choice of surgical approach depends largely on the location of the depressed articular fragments of the comminuted articular fracture that require direct reduction. Normally, we prefer to use extended approaches if there is a more central comminuted depression.

Surgical technique

The lateral tibial plateau is exposed and a submeniscal arthrotomy is performed. In cases of a pure joint depression fracture, an articular osteotomy of the lateral plateau rim is performed with an oscillating saw to allow direct visualization of the comminuted area. The lateral wall is opened, exposing the articular depression. The depressed fragments are visualized and elevated as a block to the level of the original articular surface using a wide Cobb elevator. The articular reduction is controlled both under direct visualization and fluoroscopy, and small mosaic-like fragments are corrected to their final anatomic position. Fragments are temporarily fixed with multiple 1.2-mm and/or 1.6-mm double-ended trocar tip K-wires, depending on their size. K-wires are driven through the intact portion of the tibial plateau, usually the medial region. No bone graft is used to fill the metaphyseal defect. The lateral condyle is reduced and temporarily hold in place with a periarticular reduction clamp. K-wires are advanced through the lateral condyle and the reduction clamp is removed. The reduction is monitored with fluoroscopy.

A non-locked, one-third tubular plate of appropriate length is selected according to the width of the proximal tibia at the level of the cartilage-bone interface. Using a small, sharp-edged 2.0-mm osteotome a lateral cortical window is done. Then, a sharp and thin 4.0-mm Lambotte osteotome is used to create the subchondral tunnel under fluoroscopy. During subchondral tunnel development, it is recommended to avoid forced rotational or bending movements with the osteotome into the subchondral bone. The one-third tubular plate is flattened with a hammer and introduced into the subchondral tunnel toward the medial tibial plateau, leaving at least two to three screw holes outside the lateral cortex. The plate is bent at the hole close to the lateral cortex and contoured to adequately fit the lateral tibial plateau. Plate positioning is monitored with fluoroscopy.

**Figure 1:**
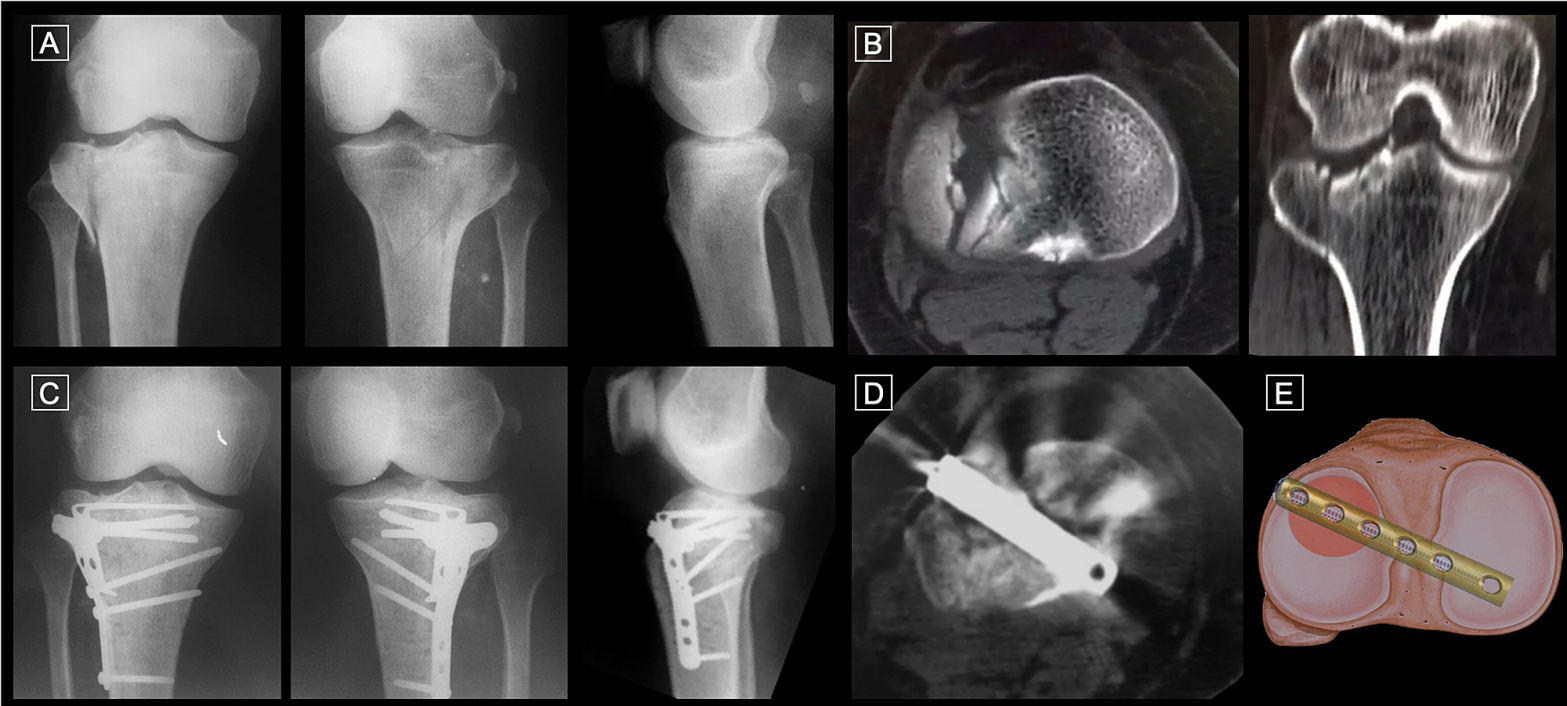
Illustration of the technique MA, 51-year-old, female patient, hit by a car A & B: Preoperative X-rays and CT scan axial and coronal cuts of the right knee showing a Kfuri-Schatzker type II A tibial plateau fracture; C & D: Postoperative X-rays and CT scan axial cut of the right knee at 12 months; E: Illustration of the technique reproducing the CT scan axial cut of the patient. CT: computed tomography

Definitive fracture fixation is carried out only after anatomic reduction has been certified. Long 3.5-mm cortical screws are placed through the bent one-third tubular plate, below its subchondral extension, in a lag-screwing technique. When necessary, a second, low-profile non-locked plate, generally a distal radius plate or another one-third tubular plate, is used to buttress the shearing fragment. Final fixation is checked with fluoroscopy (Figure 1).

Wound irrigation is done before closure. Lateral meniscus is reattached, and the wound is closed in layers. A bulky Jones-type dressing is applied to the knee joint.

Postoperative protocol

Intravenous antibiotic prophylaxis is performed for 24 hours and subcutaneous deep venous thrombosis prophylaxis (tinzaparin 3,500 IU) is given to all patients.

On the first postoperative day, the wound is checked and dressing is changed. Patients are allowed to walk using two crutches with touchdown partial weight-bearing. Standard radiographs and CT scan are performed just before discharge.

Outpatient follow-up visits are organized for two, six, and 12 weeks, six and 12 months, and then yearly thereafter, with radiographs at each visit and CT scan at 12 months. Sutures are removed in the first follow-up visit at two weeks. Weight-bearing is progressed as tolerated after six weeks, and full weight-bearing is allowed after radiological evidence of fracture healing, usually between eight to 12 weeks.

Clinical experience and results

After obtaining Institutional Review Board approval, we reviewed four consecutive patients that were managed with the above technique for fixation of tibial plateau fractures over a two-year period. There were three males and one female with a mean age of 30.7 years (range 24-51). Two fractures were classified as Kfuri-Schatzker type II A injuries, 1 as type II P injury, 1 as type III A injury [6]. All patients were operated on within 48 hours of admission. Two patients initially managed with only subchondral rafting screws only had to be re-operated on with the subchondral rafting plate technique due to a fracture malreduction observed early in the postoperative day 1. Both were re-operated on the fourth hospital day.

The mean follow-up of patients was 18 months (range 12-24). No patients were lost to follow-up. Patients had a clinical and radiological assessment at two, four, six, and 12 weeks, six and 12 months. Two patients had a clinical and radiological assessment at 24 months. All patients healed the fracture without any significant articular subsidence or loss of reduction. The range of motion (ROM) of flexion-extension for the operated knee ranged from 0° to 140°, with an average of 130°. The mean postoperative loss of flexion of the injured side compared to the uninjured knee was 10º (range 0º-20º). There was no loss of extension of the injured side compared to the uninjured knee. No complications were noted, including infection, nerve paralysis, or subchondral fragment necrosis. No patient needed implant removal after the operation.

Functional outcome was measured with the modified Lysholm scoring system [7]. The mean score at the final follow-up was 96 (range, 94-100). One patient reported joint effusion on severe exertion, and two patients were slightly impaired with squatting.

Figures 2-10 illustrate the case of a patient with a Kfuri-Schatzker type II A tibial plateau fracture, re-operated on day 4 with the subchondral rafting technique due to malreduction after the first procedure.

**Figure 2:**
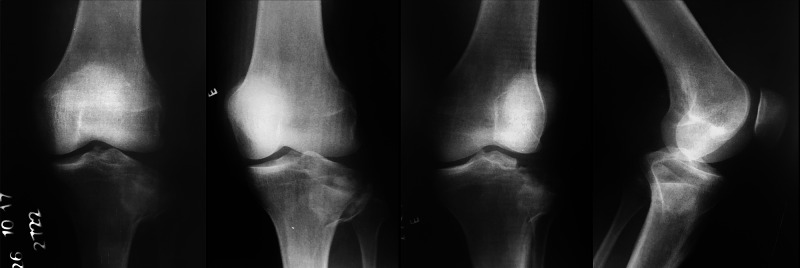
GM, 35-year-old, male patient, fall from a motorcycle Preoperative X-rays of the left knee showing a Kfuri-Schatzker type II A tibial plateau fracture

**Figure 3:**
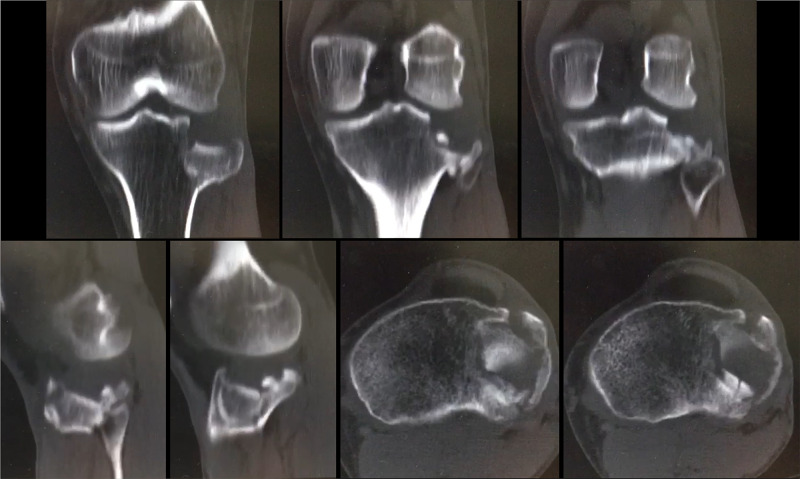
GM, 35-year-old, male patient, fall from a motorcycle Preoperative CT scan of the left knee – note the comminution at the depressed area, mainly on axial images CT: computed tomography

**Figure 4:**
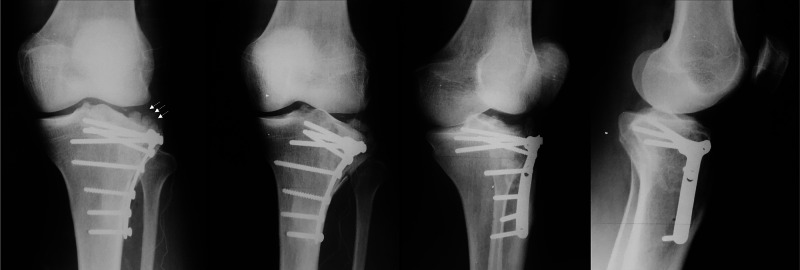
GM, 35-year-old, male patient, fall from a motorcycle Immediate postoperative X-rays of the left knee at day 2, showing malreduction of the lateral condyle of the tibial plateau – observe the secondary fragmentation that occurred during the procedure (white arrows)

**Figure 5:**
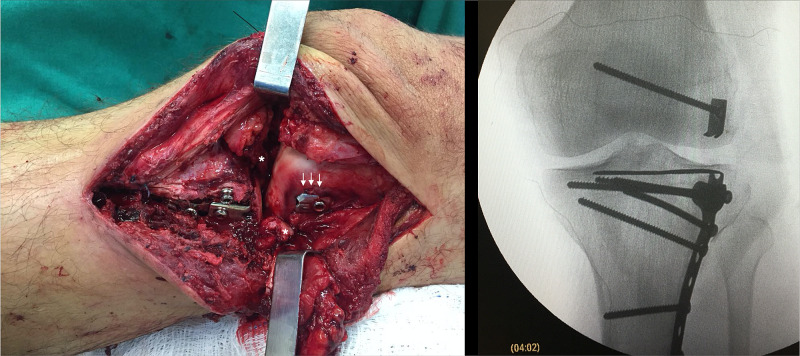
GM, 35-year-old, male patient, fall from a motorcycle Intraoperative image and fluoroscopic view of the left knee during re-operation, using an extended anterolateral approach with osteotomy of the lateral epicondyle of the femur (white arrows) and detachment of the anterior horn of the lateral meniscus (white asterisks) – note the anatomic reduction and the position of the subchondral rafting plate. The lateral femoral epicondyle was reduced to its original site and fixed with a 3.5-mm long cortical screw and a two-hole one-third tubular plate used as a spiked washer. Fixation was completed with a non-locked, long, distal radius T plate buttressing the apex of the sheared fragment of the lateral tibial plateau.

**Figure 6:**
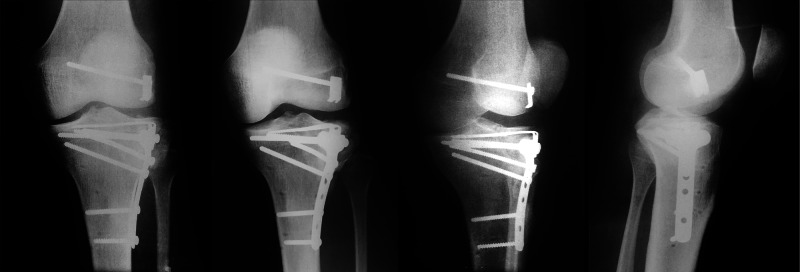
GM, 35-year-old, male patient, fall from a motorcycle Immediate postoperative X-rays of the left knee after the second operation at day 4 – anatomic reduction was obtained

**Figure 7:**
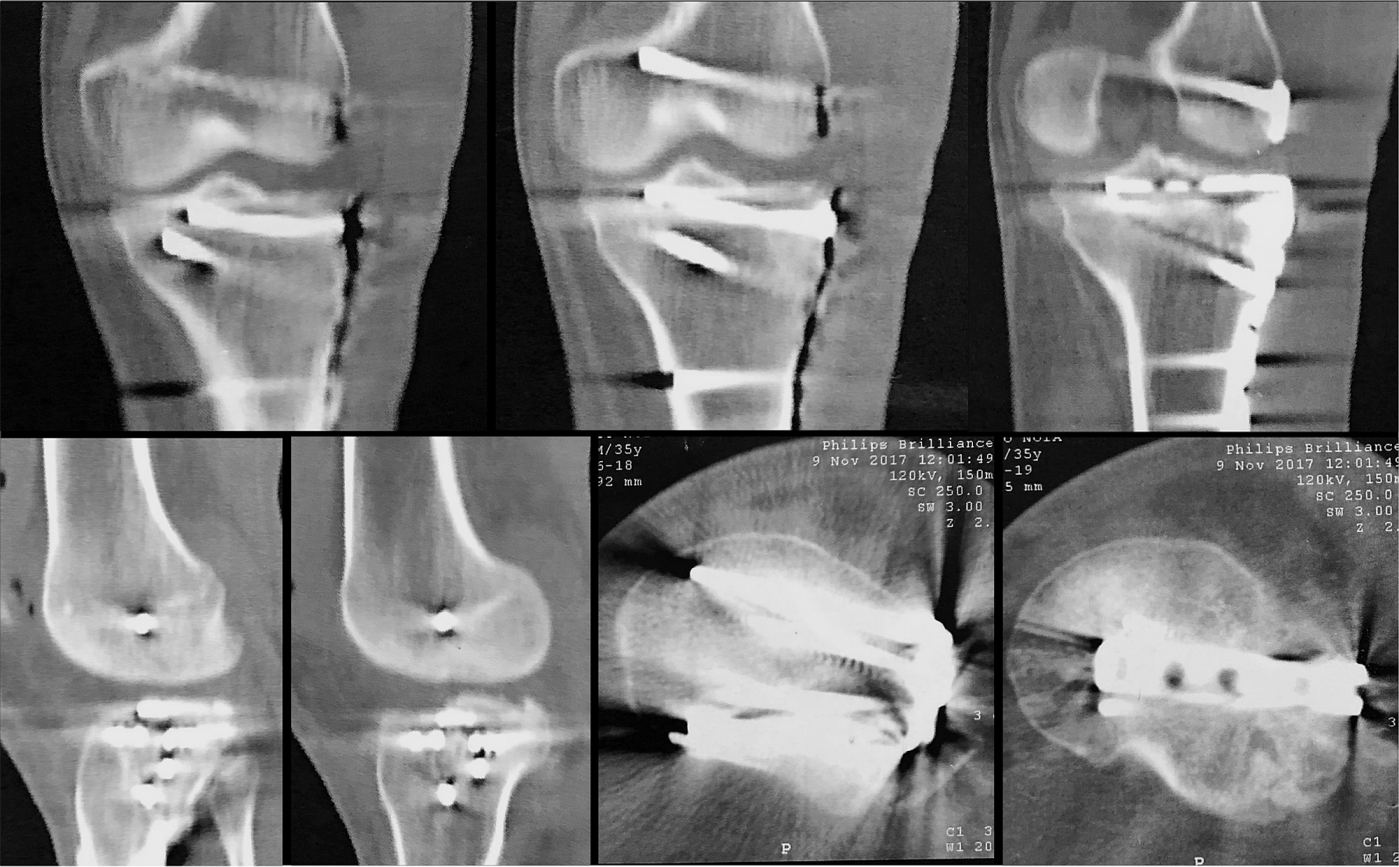
GM, 35-year-old, male patient, fall from a motorcycle Immediate postoperative CT scan of the left knee after the second operation at day 4 – anatomic reduction was obtained CT: computed tomography

**Figure 8:**
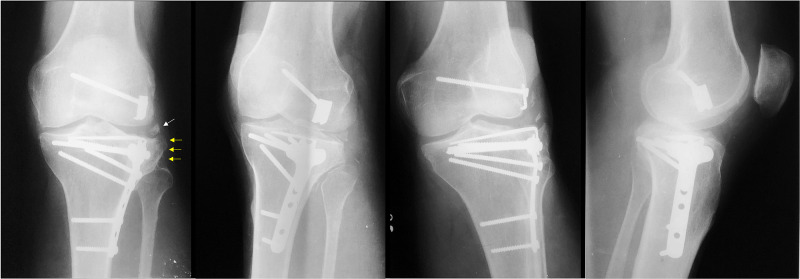
GM, 35-year-old, male patient, fall from a motorcycle Postoperative X-rays of the left knee at 24 months – there were slight peripheral subsidence and widening of the lateral tibial plateau (yellow arrows). Also, an isolated osseous fragment can be noted, which we attributed to heterotopic ossification on the lateral collateral ligament (white arrow).

**Figure 9:**
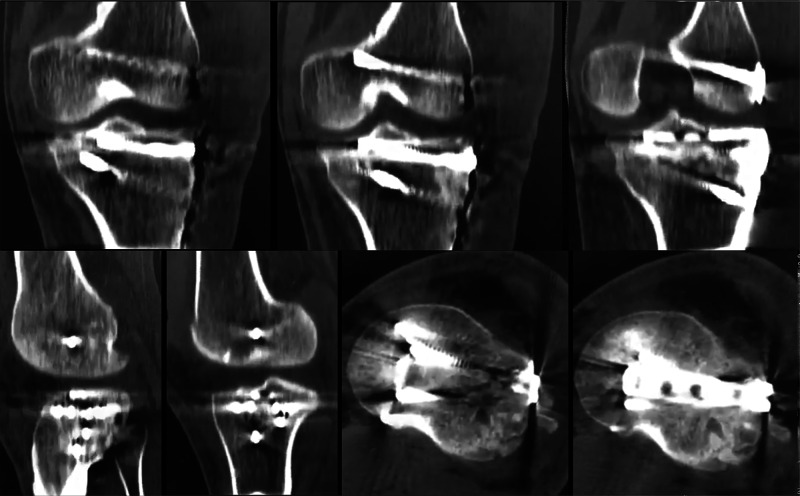
GM, 35-year-old, male patient, fall from a motorcycle Postoperative CT scan of the left knee at 24 months CT: computed tomography

**Figure 10:**
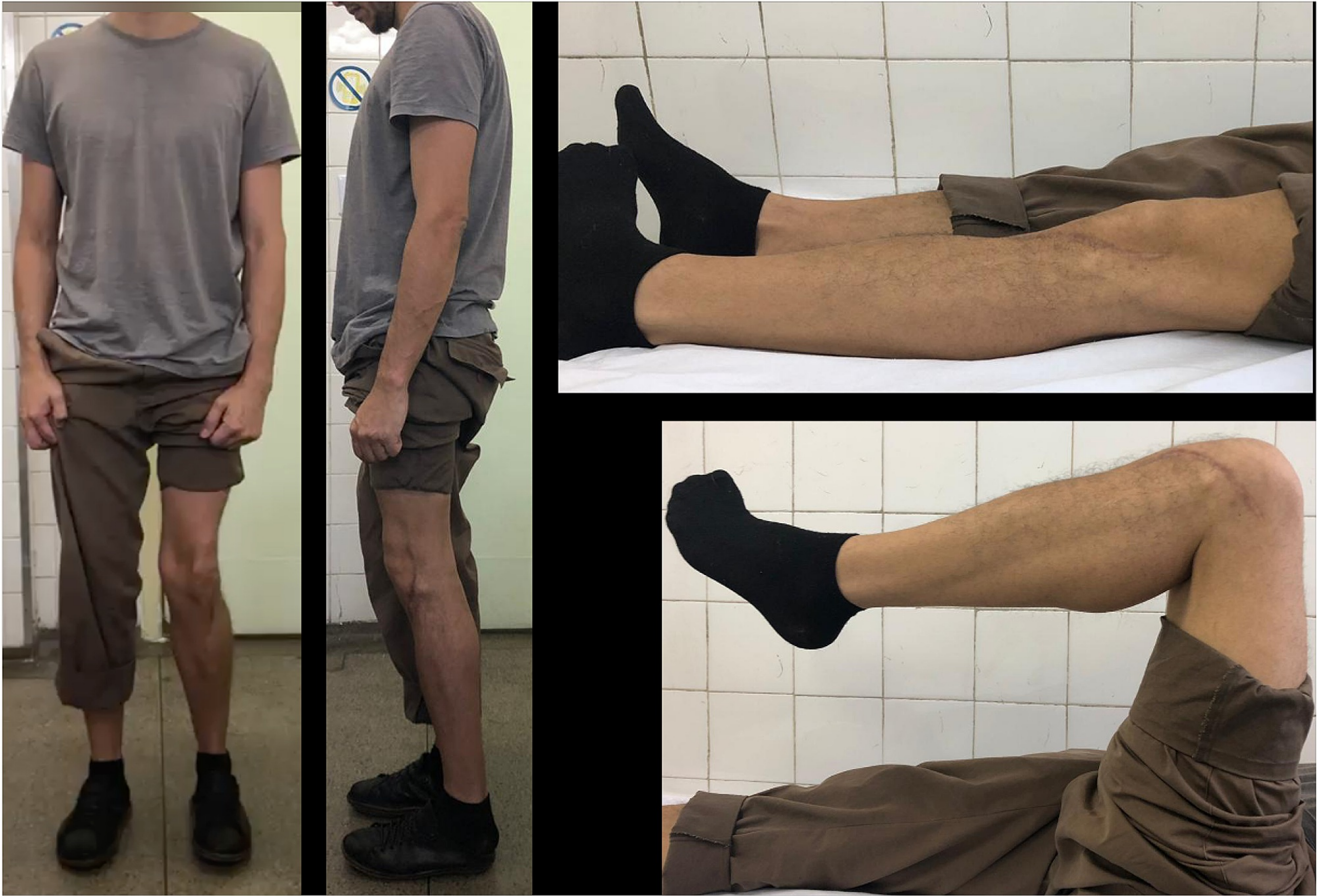
GM, 35-year-old, male patient, fall from a motorcycle Clinical pictures from the patient at 24 months, showing good knee alignment and ROM ROM: range of motion

## Discussion

Anatomical reduction of impacted articular fragments is a cornerstone concept in tibial plateau fracture reconstruction. Appropriate treatment in these cases should depend not only on the quality of reduction but also on the stable support of the subchondral fragment. Conventional treatment involves elevating the articular surface, monitored by direct, fluoroscopic, or arthroscopic vision, and subchondral rafting screw fixation [8].

However, in certain situations, such as comminuted articular depression fracture patterns, an anatomic reduction might be difficult both to obtain and maintain due to the existence of small osteochondral fragments. Traditional techniques to push up the mosaic-like fragments may result in additional fragmentation and malreduction of the articular surface. The potential benefits of inflatable bone tamps for the reduction of tibial plateau fractures, mainly in pure depression injuries, has been demonstrated [8]. Gradual inflation of the balloon has shown to provide increased reduction force on the depressed area of the tibial plateau, thus reducing pointed contact over one single small fragment [8]. In addition, the cavity volume after balloon reduction was found to be significantly smaller than the volume created by conventional bone tamping reduction [9]. In our technique, we preferred to perform a direct reduction of the depressed area so we were able to adequately establish the proper metaphyseal level for disimpaction. In our opinion, it is helpful to use the intact articular surface of the tibial plateau as a template, which facilitates lifting the impacted fragments to their anatomic position. In addition, mosaic-like fragments can be restored side-by-side. Direct visualization is not challenging in Kfuri-Schatzker type-II fractures, however, as a rule, in Kfuri-Schatzer type-III fractures with comminuted articular depression, we advise a lateral wall osteotomy. The same principles were adopted by other authors [10].

Subchondral rafting reconstruction is one of the most popular methods for preventing depression after anatomic correction of the articular surface [2-4,10]. In a biomechanical study, Karunakar et al. provided data suggesting the use of four 3.5-mm cortical screws rafting the subchondral bone [2]. Although good results had been reported with this technique, articular comminution and female patients over 65 years of age have been associated with increased articular subsidence [5]. Recently, Reul et al. proposed the use of free subchondral 2.7-mm screws to directly support the small depressed osteochondral pieces [10]. They advocated that by using screws to fix the small articular fragments, fragment fixation was improved and interfragmentary stability was increased. Nevertheless, they found only 58.8% successful reduction without significant articular step-off or anatomical malalignment at CT after three months. In our series, we noticed no significant joint surface subsidence after a minimum of 12-month follow-up. Moreover, all patients had painless function and mobility of the operated knee.

One obvious advantage of the subchondral plating technique is the ability to promote wider buttressing for the depressed osteochondral fragments. As part of the technique, we routinely flatten the plate. This facilitates its introduction into the subchondral tunnel toward the opposite region of the tibial plateau, as well as increases the footprint area and, theoretically, minimizes plastic deformation of the implant. It is known that the bending moment resistance given by the plastic moment can be significantly reduced by the effect of the concentrated transversal force when a rectangular implant is employed [11]. After setting the subchondral plating, we always place a long 3.5-mm cortical screw through the bent one-third tubular plate, below its subchondral extension, in a lag-screwing technique. Again, theoretically, this maneuver avoids major movements of the plate, acting as a modified “jail technique” [3]. In two of our cases, the technique was used as a salvage procedure for immediate lateral tibial plateau malreduction. In this difficult situation, we noticed, intraoperatively, huge fragmentation of the depressed area, which we attributed both to the original trauma and the first surgical procedure. With direct visualization of all osteochondral fragments, we were able to adequately reduce and raft the articular surface with a wider elastic implant using the subchondral plating technique.

There is a major concern that the presence of subchondral metalwork can induce microcracks in the subchondral tissues, ultimately contributing to the degeneration of the hyaline cartilage with subsequent thinning of the overlying articular cartilage [12,13]. Although mechanical overload and microdamage of the subchondral bone have been found in patients with articular cartilage damage in osteoarthritis, this seems less likely when elastic implants are placed close to the joint cartilage [14]. In an experimental sheep-model using imaging, histological, and biochemical evaluation, Goetzen et al. found no cartilage damage after subchondral screw abutment [14]. In our study, no cartilage or subchondral bone damage was observed due to the subchondral implant.

Our study has limitations. First, we present a small selected retrospective case series, lacking comparative groups with subchondral rafting constructions using screws, either with or without a plate. Opposing it, we showed consistent clinical and radiological outcomes and no complications in our sample. Second, the mechanical behavior of a flattened one-third tubular plate has not been previously studied. However, there seems to be no mechanical requirement caused by the fracture at the location where the implant is bent since we have not seen any problems related to this. It has been shown that stainless steel implants can undergo a great amount of plastic deformation before reaching the point of ultimate failure and can tolerate more shape change before breaking [15]. Therefore, we do not believe it can be seen as a disadvantage of the technique. Third, there is a potential risk of intraoperative iatrogenic fracture of the already fractured and weakened lateral wall of the condylar fragment during both subchondral tunnel development and insertion of the flattened one-third tubular plate. Therefore, we strongly advise to start the cortical cut using a 2.0-mm sharp and thin Lambotte osteotome and then to use a 4.0-mm Lambotte osteotome to create the subchondral tunnel. Moreover, it is recommended to avoid forced rotational or bending movements with the osteotome into the subchondral bone. Finally, we did not use bone graft in the metaphyseal area to support the subchondral articular fragments. Despite the beneficial biomechanical effects of bone void fillers, and especially calcium phosphate cement [8,16,17], clinical studies demonstrated that the use of bone graft or substitutes do not affect subsidence at a statistically significant level [1,4,5]. Interestingly, it appears the position in which subchondral implants are placed is more important in maintaining articular reduction [5]. Nevertheless, theoretically, in severe articular surface comminution with a large impaction area anterior to posterior, the diameter of the one-third tubular plate can be not wide enough to support all the affected area. We didn’t have any case like this, but it seems beneficial in this situation to apply the subchondral rafting plate supporting the larger affected articular area and to fill the unsupported subchondral defect with a bone substitute, especially calcium phosphate cement [8,16,17].

## Conclusions

In summary, our preliminary results show that this novel subchondral rafting plate technique is a potentially safe and cost-effective strategy to be incorporated in the daily practice of the orthopedic trauma surgeon, especially in certain challenging circumstances when a salvage procedure is required due to lateral tibial plateau fracture malreduction and anatomically designed locking plates are not available. Further comparative studies with a larger sample of patients and longer follow-up are necessary to evaluate the real benefits of this promising technique.
